# A Propensity-Matched Comparison of Ischemic Brain Lesions on Postprocedural MRI in Endovascular versus Open Carotid Artery Reconstruction

**DOI:** 10.3390/jcdd10060257

**Published:** 2023-06-13

**Authors:** Zsuzsanna Mihály, Samuel Booth, Dat Tin Nguyen, Milán Vecsey-Nagy, Miklós Vértes, Zsófia Czinege, Csongor Péter, Péter Sótonyi, Andrea Varga

**Affiliations:** 1 Department of Vascular and Endovascular Surgery, Heart and Vascular Center, Semmelweis University, 1122 Budapest, Hungary; zsuzsannamihaly@gmail.com (Z.M.); samuel.booth97@gmail.com (S.B.); czinege.zsofia@gmail.com (Z.C.); sotonyi@hotmail.com (P.S.); 2 Department of Cardiology, Heart and Vascular Center, Semmelweis University, 1122 Budapest, Hungary; nguyen.tindatdat@gmail.com (D.T.N.); vnagymilan@gmail.com (M.V.-N.); petercsongor@yahoo.com (C.P.); 3 Hungarian Vascular Radiology Research Group, 1122 Budapest, Hungary; 4 Department of Interventional Radiology, Heart and Vascular Center, Semmelweis University, 1122 Budapest, Hungary; vertesmiki@gmail.com

**Keywords:** carotid artery stenosis, carotid artery stenting, carotid artery endarterectomy, ischemic brain lesions, carotid plaque features, ct angiography, cardiovascular imaging

## Abstract

(1) Study purpose: The aim of our prospective single-center, matched case–control study was to compare the number and volume of acute ischemic brain lesions following carotid endarterectomy (CEA) versus carotid artery stenting (CAS) using a propensity-matched design. (2) Methods: Carotid bifurcation plaques were analyzed by using VascuCAP software on CT angiography (CTA) images. The number and volume of acute and chronic ischemic brain lesions were assessed on MRI scans taken 12–48 h after the procedures. Propensity score-based matching was performed at a 1:1 ratio to compare the ischemic lesions on postinterventional MR. (3) Results: A total of 107 patients (CAS, *N* = 33; CEA, *N* = 74) were included in the study. There were significant differences in smoking (*p* = 0.003), total calcification plaque volume (*p* = 0.004), and lengths of the lesion (*p* = 0.045) between the CAS and CEA groups. Propensity score matching resulted in 21 matched pairs of patients. Acute ischemic brain lesions were detected in ten patients (47.6%) of the matched CAS group and in three patients (14.2%) in the matched CEA group (*p* = 0.02). The volume of acute ischemic brain lesions was significantly larger (*p* = 0.04) in the CAS group than in the CEA group. New ischemic brain lesions were not associated with neurological symptoms in either group. (4) Conclusions: Procedure-related new acute ischemic brain lesions occurred significantly more frequently in the propensity-matched CAS group.

## 1. Introduction

It is common to observe new ischemic brain lesions after carotid artery stenting (CAS), with an incidence rate as high as 40%. In comparison, carotid endarterectomy (CEA) is associated with a lower rate of 12% [1]. Rots and colleagues in their systematic review identified the predictors of new ischemic brain lesions after CEA as prior neurological events, impaired cerebrovascular reserve capacity, and higher levels of inflammatory markers. While older age, plaque vulnerability, and complex anatomy of the carotids and aortic arch were established as predictors of CAS-related ischemic brain lesions [1]. 

For nearly a decade, it has been widely recognized that diffusion-weighted imaging (DWI) positive lesions, which indicate acute ischemic brain injury, are linked to an increased risk of recurrent cerebrovascular events [2,3]. However, randomized clinical trials have not provided evidence regarding a potential connection between cognitive impairment and silent ischemic brain lesions [4] Nevertheless, results from the Neurovision study [3] and ICSS study [5] proposed that silent ischemic brain lesions may lead to cognitive impairment. Furthermore, a prospective longitudinal study has demonstrated that embolic infarct volumes correlate with long-term cognitive changes [6]. These findings support the notion that microembolization should be considered as a surrogate measure for carotid disease management.

Most previous studies reporting new DWI-positive lesions after carotid reconstruction (CAS or CEA) were either retrospective or had varied selection criteria for the open versus endovascular approach. These criteria may have been based on individual patient characteristics, such as age and comorbidities, potentially leading to selection bias [1]. While intervention-related differences, such as complex aortic arch anatomy, shunt use, or lack of embolic protection devices cannot be entirely eliminated, proper study design can help minimize bias pertaining to patient selection and plaque characteristics.

To the best of our knowledge, this is the first investigation to focus on the risk factors for DWI-positive lesions after CEA and CAS, with comparable patient and plaque characteristics in the two cohorts. Our aim is to determine and compare the incidence and volume of procedure-related new ischemic brain lesions in patients undergoing CAS and CEA after eliminating differences in patient and plaque characteristics between the groups using a propensity-matched design.

## 2. Materials and Methods

From 1 January 2019 to 31 January 2021, data were prospectively collected yielding 74 CEA and 33 CAS patients. All patients provided written informed consent prior to the examination. The study was approved by the institutional ethical committee (IV/667-1/2022/EKU) and was carried out in accordance with the Declaration of Helsinki. (SE-KREB 84/2019). Inclusion and exclusion criteria are summarized in Table 1.

### 2.1. Patients’ Enrollment

This was a prospective, single-center, matched case–control study. Hypertension was defined in accordance with the American Heart Association (AHA) guidelines [7], while diabetes mellitus and dyslipidemia were defined by the European Society of Cardiology (ESC) guidelines [8,9]. Patients who currently smoke were placed into the smokers’ group. After CT angiography (CTA), all patients were recommended to receive the best medical therapy, which consisted of aspirin 100 mg or clopidogrel 75 mg, along with a statin, based on the European Society for Vascular Surgery (ESVS) guideline [4]. Symptomatic carotid artery stenosis (defined as a relevant neurological event within 6 months) was defined according to the ESVS guideline as well [4]. 

### 2.2. CTA Protocol

Patients underwent carotid CTA as part of standard-of-care diagnostic evaluation using a routine protocol using a 256-slice scanner (Brilliance iCT 256, Philips Healthcare, Best, The Netherlands). The evaluation included a non-contrast brain CT followed by CTA of the carotid arteries from the aortic arch to the vertex, with bolus tracking. The following imaging parameters were used for data acquisition: 120 kV, 50–160 mAs/slice, and a slice thickness of 0.67 mm, with Philips^®^ IMR reconstruction. Intravenous contrast agent, 50 mL Iomeron400, was injected at a flow rate of 5 mL/s.

Carotid stenosis severity was evaluated through CTA as part of routine clinical practice based on NASCET criteria [10]. The stenosis location (bifurcation or suprabulbar) and plaque calcification (extent and thickness) were determined by consensus reading by ZM and ZC. The extent of plaque calcification was measured using a 5-point scale described by Babiarz et al. [11].

The pre-procedural CTA was obtained, and quantitative plaque characteristics were assessed using commercially available atherosclerotic plaque image analysis software (VascuCAP Build A.3 25 January 2021 12:22:43; Elucid Bioimaging, Wenham, MA, USA). The automatic centerline identification was pre-tested in the VascuCAP software.

#### Quantitative Plaque Analysis

**Figure 1:**
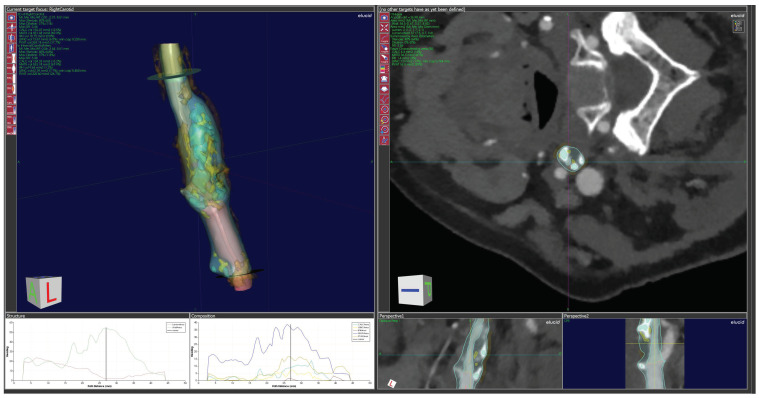
After morphologic segmentation of the lumen and wall in VascuCap and selection of the plaque causing the stenosis, the software-generated 3D model and the longitudinal and cross-sectional image of the lesion are shown in a CTA image.

Following automatic segmentations with the VascuCAP software, manual refinement was applied to correct the boundaries at the reconstructed surface in the lumen and wall. Past the bifurcation, the external carotid artery was excluded from the study by delineating a surface perpendicular to its axis. After the semi-automatic segmentation and vascular reconstruction had been performed, the entire artery section’s plaque geometry and tissue composition were automatically computed. Figure 1 shows an example of plaque analysis.

Quantitative metrics were computed for matching in our study, which included the following: calcification volume (CALCVol), intraplaque hemorrhage volume (IPHVol), lipid-rich necrotic core volume (LRNCVol), fibrous tissue matrix volume (MATXVol), length, maximum remodeling ratio, and maximum stenosis by area. CALCVol, IPHVol, LRNCVol, and MATXVol refer to the calcified, IPH, LRNC, and MATX volume in the plaque and they were measured in millimeters cubed (mm^3^). The plaque length corresponds to the length in millimeters (mm), of the atherosclerotic plaque in the ICA and/or CCA. The maximum remodeling ratio is defined as the maximum wall remodeling ratio, based on software calculation, which is the cross-sectional lumen area to wall area ratio. The maximum stenosis by area is the maximum cross-sectional stenosis of the target artery based on area.

### 2.3. Procedures 

Our vascular team, which included interventional radiologists, vascular surgeons, and consultant neurologists, carefully considered treatment options based on the current ESVS guidelines. Treatment options were carefully considered by our vascular team, including interventional radiologists, vascular surgeons, and consultant neurologists based on the current ESVS guideline [4].

#### 2.3.1. CEA Protocol 

General anesthesia was used for all surgical carotid artery reconstructions, which were performed by five experienced vascular surgeons. Most patients underwent eversion endarterectomy, while selective shunting was performed using the PRUITT Inahara^®^ Carotid shunt (LeMaitre Vascular Inc., Burlington, MA, USA) with conventional endarterectomy and closure by bovine patch angioplasty. After the operation, all patients underwent a basic neurological evaluation and were discharged on postoperative day three. Single antiplatelet therapy was prescribed unless there was a cardiac indication for longer dual antiplatelet therapy.

#### 2.3.2. CAS Protocol 

Interventions were carried out using the Wallstent self-expanding stent (Boston Scientific Corp., Marlborough, MA, USA) or with Roadsaver^®^ Carotid artery stent system (Terumo Corp, Tokyo, Japan) by four experienced interventional radiologists through radial or common femoral artery approach. In all cases, embolic protection was provided using the Filterwire EZ embolic protection device (Boston Scientific, Marlborough, MA, USA). In all cases, the shortest balloon or stent that could cover the entire lesion was chosen. The punctured arteries were compressed manually or sealed with a closure device. After the intervention, all patients underwent a basic neurological evaluation and were discharged on postoperative day one. Patients were prescribed dual antiplatelet therapy for up to 1 month, followed by lifelong single antiplatelet therapy, unless a cardiac indication required longer dual antiplatelet therapy.

### 2.4. Postoperative MR 

Non-contrast brain MRI was performed on both CAS and CEA patients 12–48 h after the procedures using a Siemens Magnetom Aera 1.5T scanner (after May 2019) or a Philips Achieva 1.5T scanner (before May 2019) with standard head array coils. The MRI protocol for CAS patients included DWI, fluid-attenuated inversion recovery (FLAIR), T2*-gradient echo (T2*-GRE—before April 2019) or susceptibility-weighted imaging (SWI after 2019) sequences, while that of the CEA patients included diffusion tensor imaging (DTI), FLAIR, T2*-GRE or SWI sequences. The acquisition parameters are specified in Table 2.

The presence, number, and distribution (cortical/subcortical, deep white matter, basal ganglia, or watershed) of recent ipsilateral and contralateral acute ischemic lesions detected on DWI were registered. The volume of each lesion was calculated by summing the products of the manually measured region of interest area and the slice thickness. If a patient had more than one lesion, the total volume of all lesions in each hemisphere was added.

The Fazekas scale [12] was used to quantify the white matter FLAIR hyperintense chronic lesions in both the ipsilateral and contralateral hemispheres. The presence, number, and distribution of chronic infarcts were registered similarly to the DWI-positive acute ischemic brain lesions. The assessing radiologist was blinded to clinical data. Two radiologists (CP with 3 years of experience and AV, a board-certified neuroradiologist with 18 years of experience) performed the post-procedural MR evaluation to determine the presence of new and chronic ischemic brain lesions.

### 2.5. Statistical Analysis 

Continuous variables were expressed in median and interquartile ranges, while categorical variables as numbers and percentages. Continuous data were compared between groups using the Mann–Whitney U test, while the Fisher exact test was used to assess differences between categorical variables. Given the significant differences in the distribution of key variables between the study groups, propensity score matching was used to ensure a balance of covariates. 

Propensity score matching was used to compare outcomes between patients treated with CAS and CEA, with matching performed in a 1:1 ratio. Propensity score refers to the conditional probability of a specific treatment assignment based on a set of measured baseline covariates. To ensure appropriate propensity matching of the cohorts, all baseline characteristics were included in the calculation of propensity scores that were considered potentially relevant in the decision-making process. These factors included demographic parameters and cardiovascular risk factors such as age, sex, hypertension, diabetes mellitus, dyslipidemia, current smoking, previously documented coronary artery disease, and the location of the carotid plaque. Additionally, quantitative plaque parameters, including the total calcified plaque volume, the volume of intraplaque hemorrhage, lipid-rich necrotic core and matrix, the length of the lesion, the remodeling ratio, and the degree of area stenosis, were incorporated into the model. Thereafter, these factors were used to generate a propensity score in both cohorts. The propensity score was then used to perform propensity score matching using the method of nearest matching with a 0.2 caliper width. 

Binary logistic regression analysis was used to compare MRI outcome measures between the two treatment groups, and a two-sided *p*-value of <0.05 was considered significant for all analyses. All statistical calculations were conducted in the R environment (v. 4.0.0).

Patient baseline characteristics, as well as plaque location (whether at the bifurcation or suprabulbar), were compared using the Fisher Exact Test before and after propensity score matching. For the quantitative variables obtained from the VascuCAP analysis, the Mann–Whitney U test was used.

## 3. Results

The study included a total of 107 carotid interventions that met the required inclusion and exclusion criteria, comprising 74 CEAs and 33 CAS. None of the patients in the CAS group were symptomatic. Out of seventy-four CEA patients, only five had experienced a neurologic event related to carotid stenosis (two strokes and three TIAs) within 6 months before the operation. Nine right-sided CAS lesions were treated using common femoral artery (CFA) access while eight were treated via radial artery access. Two left-sided CAS lesions were treated using CFA access and fourteen via radial artery access.

### 3.1. Patients’ Characteristics and Propensity-Matched Pairing

Baseline characteristics, as well as qualitative and quantitative carotid plaque analysis results, were collected for all 107 patients included in the study. There were no significant differences in the baseline patient characteristic except smoking (*p* = 0.003) between the CAS and CEA groups, but there were significant differences in plaque characteristics (total calcification volume *p* = 0.004 and lesion length *p* = 0.045). Table 3 and Table 4 summarize the patient and plaque characteristics of the full patient cohort. 

Due to significant differences in key characteristics between the two groups, CEA patients were matched with CAS patients using a propensity score. This permitted the creation of a well-balanced cohort with regard to baseline covariates. A total of 21 appropriate pairs were identified, with no significant differences in baseline characteristics. Among the 21 CEA patients, 2 were previously symptomatic with TIAs. 

### 3.2. Postprocedural MR Imaging

**Figure 2:**
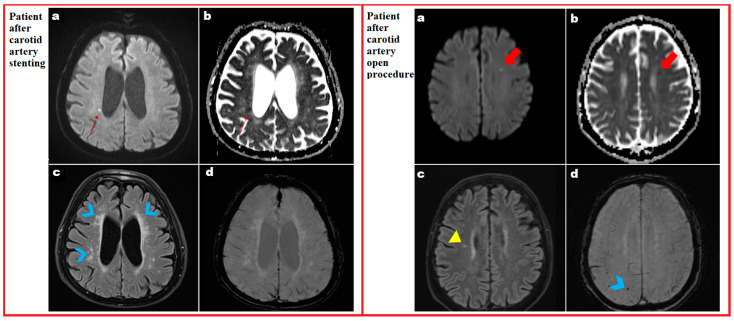
MR images of a CAS and a CEA patient. (**Left side**), Panel a: Right periventricular new ischemic focus with high signal intensity on DWI trace image after CAS (red arrow). Panel b: Corresponding low signal intensity on ADC map (red arrow). Panel c: Periventricular (and subcortical) FLAIR hyperintensities on both sides suggestive of severe chronic small vessel disease (blue arrows). Panel d: No signal void on SWI suggestive of a hemorrhagic component, if present. (**Right side**), Panel a: Left frontal periventricular new ischemic focus with high signal intensity on DWI trace image following CEA (red arrow). Panel b: Corresponding low signal intensity on ADC map (red arrow). Panel c: Mild small vessel disease presenting as FLAIR hyperintensities (yellow arrow). Panel d: Right parietal chronic micro-hemorrhage with SWI signal void (blue arrow).

The 21 matched pairs showed a significant difference in the presence of new DWI lesions [CAS: 10/21 (47.6%) versus CEA: 3/21 (14.2%); *p* = 0.02), as well as in the volume of new lesions [CAS: 0.0 (0.0–45.3) mm^3^ versus CEA: 0.0 (0.0–0.0) mm^3^; *p* = 0.04. Table 5 shows the results of the detailed brain MR evaluation. The volume of chronic ischemic brain lesions did not differ significantly between the two groups. No perioperative neurological events were noted in the groups. One of the patients with symptomatic carotid stenosis had a DWI-positive lesion after CEA. Figure 2 provides an impression of typical lesion localization and size in postprocedural MR images. 

### 3.3. Predictors of New Postprocedural Cerebral Ischemic Lesions in the Propensity-Matched CEA or CAS Patients

In the propensity-matched study population, the volume of chronic infarcts was a predictor for a higher volume of new ischemic lesions (0.01 [ 95% CI 0.001–0.02] *p* = 0.02), while the Fazekas scale, presence, and localization of chronic infarcts were not, as it is presented in Table 6. 

## 4. Discussion

Most previous studies comparing the impact of CAS and CEA in acute ischemic brain lesions were retrospective or prospective without propensity-matching. Our results confirm the findings of previous large studies, but the use of propensity-matched cohort design is unique compared to other studies. Treatment selection bias can be minimized through propensity score matching. The odds of procedure-related embolism are dependent on the presence of comorbidities or plaque characteristics related to embolization, and each risk type should be documented to create a proper risk prediction model and to choose an adequate method of revascularization. 

Silent ischemic infarction is one of the criteria used in asymptomatic patients to categorize them as “high stroke risk,” making it an indication for revascularization [4]. A systematic meta-analysis of carotid revascularization studies provided a correlation between the occurrence of silent DWI-positive lesions and clinically manifested stroke [13]. Based on these findings, a different strategy should be utilized for patients with chronic cerebral lesions. 

In a multicenter prospective non-randomized study, increasing age and obesity were identified as independent risk factors for larger volumes of silent brain infarcts after CAS, while diabetes mellitus was identified as a risk factor after CEA. The study found a positive correlation between age and infarct volume in CAS and a negative correlation in CEA. The authors explained these conflicting results by pointing out differences in plaque features between CAS and CEA [14]. The correlation between diabetes mellitus and larger infarct volumes following CEA could not be explained by diabetes mellitus-related intracranial disease, and it may have been due to bias as diabetes mellitus management is known to be suboptimal in CAS [13]. In our study, propensity-matching was used to eliminate and avoid treatment selection bias based on patient characteristics and/or plaque features. 

A single-center study found that short, calcified lesions were an independent risk factor for new DWI lesions after CAS. In that study, lesion length was classified as less or more than 2 cm long, and calcification was classified as heavy or mild on ultrasound [15]. In our study, we eliminated both calcification type and lesion length as potential biases. We defined plaque characteristics quantitatively using VascuCap software, which is more precise than dichotomous categorization of calcification/plaque length with consensus reading only. Plaque calcification features did not differ between our CAS and CEA groups either. Therefore, plaque features should be defined as independent risk factors for microembolization only if measured quantitatively and not categorically on carotid CTA in publications. Recent software developments facilitate the quantitative assessment of plaque volume or burden on coronary CTA, and the identification of subtypes of plaque based on their attenuation density [16]. We recommend that quantitative carotid plaque assessment is used not just in research, but in clinical use as well, as coronary CTA has been proven effective for the serial quantitative assessment of coronary artery disease progression [17].

The previous publications showed a higher number of new DWI lesions on 3T than 1.5T after carotid revascularization [18], which can be the reason for the relatively low incidence of DWI-positive lesions in our study group. In accordance with previously published prospective studies [6,14], we observed a significant difference in the volume of new ischemic lesion volume between CAS and CEA patients. The use of semi-automatic DWI lesion volume analysis is less time-consuming and more precise than the classification of absence/presence. However, in our study, we utilized manual DWI segmentation. The ICSS study demonstrated that age-related white matter changes are predictors for higher perioperative stroke risk in CAS, but not in CEA [19]. In our study cohort, ipsilateral deep white matter lesions did not have a predictive value for either the presence or volume of new DWI lesions, possibly due to the relatively low number of enrolled patients. 

Notably, we observed no periprocedural neurological events, which may be attributed to the relatively low number of cases, our high-volume tertiary single center, and the use of embolic protection devices during CAS or selective shunting under CEA. Furthermore, the bias could be that clinical assessment for procedural complications was not performed by an independent neurologist; only a basic neurological evaluation was performed. However, the average risk of procedural stroke in studies that reported neurologist confirmation of the event was only slightly higher than in studies that did not, so this bias may be negligible [13]. The Neurovision study has suggested that perioperative covert stroke may play an important role in explaining cognitive decline following non-cardiac surgery based on neurocognitive testing [20], perhaps neurocognitive testing may provide a differential outcome in the postprocedural neurological evaluation in patients with new DWI lesions. 

There are several limitations to this study. Firstly, it is a non-randomized study. Secondly, the sample size is limited, and the number of events is low. Thirdly, preoperative MR scans were not obtained for CAS patients, therefore the postprocedural MR could not be compared to preprocedural MR. Fourthly, there was heterogeneity regarding the procedures with CAS performed either from radial or femoral access using two different brands of stents, and the selective shunt use for CEA was based on the individual decision of the surgeons. In the modern era, a further limitation is that the study did not include any transcarotid artery stenting cases. Despite these limitations and the inherent procedure-related bias of our study, we believe that our results add to the existing knowledge and provide a good level of evidence for the paucity of data in the literature. 

## 5. Conclusions

Our study suggests that using quantitative carotid plaque assessment is a superior way to identify predictors for periprocedural new ischemic brain lesions, as it was more sensitive than using dichotomous qualitative plaque feature descriptions. After propensity matching was employed to eliminate differences in patient and plaque characteristics, the CAS group showed a significantly higher appearance and volume of procedure-related new ischemic DWI lesions. However, new ischemic brain lesions were not associated with neurological symptoms in either group. We propose that the higher occurrence of periprocedural microembolization is related to endovascular manipulations in the supraaortic vessels during CAS, while the observed differences cannot be accounted for by patient or plaque characteristics. 

## Figures and Tables

**Table 1:** Inclusion and exclusion criteria of the study.

Inclusion Criteria	Exclusion Criteria
Signed informed consent form	Age under 50 years
Significant atherosclerotic stenosis of the extracranial internal carotid artery	Restenosis or radiation therapy induced carotid artery stenosis
Previous carotid CT angiography (performed according to institutional protocol)	Neurological event 15 days before reconstruction
	End stage chronic kidney disease (Stage V)
	Pacemaker or ICD implantation
	Atrial fibrillation

**Table 2:** MRI protocol parameters for carotid artery stenting and carotid endarterectomy patients. DTI = diffusion tensor imaging; FLAIR = fluid-attenuated inversion recovery; T2*-GRE = T2*-gradient echo, SWI = susceptibility-weighted imaging * Siemens Magnetom Aera 1.5T, ** Philips Brilliance 1.5T.

MRI Sequence Parameters	DTI **	DTI *	DWI *	DWI **	FLAIR **	FLAIR *	SWI **	T2*-GRE *
Orientation	axial	axial	axial	axial	axial	axial	axial	axial
Repetition time (TR) (ms)	4300–5600	14,000–29,000	3607–3760	9000–9300	7000–8500	6000	48	890–1023
Echo time (TE) (ms)	83	62	87	88	97	120	40	23
Flip angle (FA) (degree)	90	90	90	90	150	90	15	18
Turbo factor	48	59	37	71	25	23	1	1
Inversion time (TI) (ms)	-	-	-	-	2440	2000	-	-
Field of view (FOV) (mm)	230 × 230	230 × 230	230 × 230	230 × 230	230–240 × 230–240	230 × 230	205–220 × 235–240	230 × 230
Number of excitations	1	1	2	1	2	2	1	2
Slice thickness/ spacing (mm)	5/6.5	2/2	4/5	4/5	4/5	4/5	2.2/2.2	4/5
Matrix	128 × 128	128 × 128	128 × 128	128 × 128	288 × 288	288 × 288	248–348 × 288–384	256–256
Encoding directions	20	32	3	3	-	-	-	-
B values (s/mm^2^)	0 and 1000	0 and 1000	0 and 1000	0 and 1000	-	-	-	-

**Table 3:** Baseline patient characteristics before and after propensity matching.

Patients’ Characteristics	Before Propensity Matching (*N* = 107)			21 Propensity Matched Pairs (*N* = 42)		
	CAS *N* = 33	CEA *N* = 74	*p*	CAS *N* = 21	CEA *N* = 21	*p*
Sex (female)	13 (39.4%)	29 (39.2%)	0.98	6 (28.6%)	10 (47.6%)	0.34
Age (years)	69 (62–74.5)	69 (65–74.25)	0.39	70 (65–76.5)	67.5 (65.75–72.75)	0.58
Hypertension	29 (88%)	68 (92%)	0.51	17 (81%)	19 (90%)	0.66
Diabetes mellitus	7 (21%)	29 (39%)	0.07	7 (33%)	3 (14%)	0.28
Smoking	20 (60.6%)	22 (29.7%)	0.003	9 (42.9%)	6 (28.6%)	0.52
Dyslipidemia	27 (81.8%)	51 (68.9%)	0.17	17 (80.95%)	16 (76.2%)	1.00
Coronary artery disease	12 (36.4%)	20 (27.0%)	0.33	8 (38.1%)	6 (28.6%)	0.74

**Table 4:** Baseline plaque characteristics before and after propensity matching.

Plaque Characteristics	Before Propensity Matching (*N* = 107)			21 Propensity Matched Pairs (*N* = 42)		
	CAS *N* = 33	CEA *N* = 74	*p*	CAS *N* = 21	CEA *N* = 21	*p*
Qualitative features of carotid plaques						
Plaque location (bifurcation or suprabulbar)	31 (93.9%)	67 (90.5%)	0.560	19 (90.5%)	18 (85.7%)	1.000
	2 (6.1%)	7 (9.5%)		2 (9.5%)	3 (11.1%)	
Plaque calcification score extent (0–4)	1 (3.0%)	3 (4.1%)	0.231	0 (0%)	1 (4.8%)	0.561
	4 (12.1%)	14 (18.9%)		2 (9.5%)	4 (19.0%)	
	13 (39.4%)	14 (18.9%)		7 (33.3%)	5 (23.8%)	
	9 (27.3%)	30 (40.5%)		7 (33.3%)	6 (28.6%)	
	6 (18.2%)	13 (17.6%)		5 (23.8%)	5 (23.8%)	
Plaque calcification score thickness (0–4)	1 (3.0%)	2 (2.7%)	0.744	0 (0%)	1 (4.8%)	0.654
	5 (15.2%)	13 (17.6%)		2 (9.5%)	2 (9.5%)	
	13 (39.4%)	27 (36.5%)		8 (38.1%))	9 (42.9%)	
	11 (33.3%)	18 (24.3%)		8 (38.1%)	4 (19.0%)	
	3 (9.1%)	14 (18.9%)		3 (14.3%)	5 (23.8%)	
Quantitative features of carotid plaques						
Total volume of intraplaque hemorrhage (mm^3^)	0 (0–2.8)	0.36 (0–2.14)	0.357	0.05 (0–4.70)	0.44 (0–10.23)	0.644
Total volume of lipid-rich necrotic core (mm^3^)	98.5 (31.7–165.5)	128.35 (50.14–200.50)	0.761	119.08 (37.85–207.48)	65.88 (27.26–176.88)	0.263
Total volume of matrix (mm^3^)	406.4 (298.3–676.1)	504.46 (358.1–777.2)	0.640	439.49 (330.09–760.11)	498.68 (332.78–853.55)	0.910
Total calcified plaque volume (mm^3^)	34.4 (19.6–90.30)	86.1 (45.5–159.0)	0.004	40.9 (27.4–91.1)	52.2 (25.2–188.4)	0.596
Remodeling ratio	2.44 (1.85–3.06)	2.33 (1.51–3.80)	0.968	2.58 (2.08–3.22)	1.73 (1.26–3.29)	0.080
Degree of area stenosis	82.4% (64.3–89.1)	79.9% (69.1–89.2)	0.964	87.0 (61.7–91.7)	77.25 (68.2–90.87)	0.792
Length of lesion (mm)	24.7 (18.9–32.8)	28.8 (22.7–37.4)	0.045	26.4 (20.8–32.9)	27.5 (19.8–36.4)	0.936

**Table 5:** Comparison of new and chronic cerebral lesions between the groups.

	CAS	CEA	*p*
	*N* = 21	*N* = 21	
New DWI lesion appeared (yes)	10 (47.6%)	3 (14.3%)	0.02
New DWI lesion localization			
None	11 (52.4%)	18 (85.7%)	0.08
Ipsilateral	6 (28.6%)	3 (14.3%)	
Contralateral	3 (14.3%)	0	
Bilateral	1 (4.7%)	0	
DWI lesion volume (mm^3^)			
Total	0.0 (0.0–45.3)	0.0 (0.0–0.0)	0.04
Ipsilateral	0.0 (0.0–31.6)	0.0 (0.0–0.0)	0.14
Contralateral	0.0 (0.0–0.0)	0.0 (0.0–0.0)	0.07
Fazekas scale [12]			
Ipsilateral	1.0 (0.0–2.0)	1.0 (0.0–2.0)	0.35
Contralateral	1.0 (0.5–1.5)	1.0 (0.0–2.0)	0.34
Chronic infarct volume (mm^3^)			
Total	0.0 (0.0–1379.3)	0.0 (0.0–10.4)	0.18
Ipsilateral	0.0 (0.0–26.0)	0.0 (0.0)	0.44
Contralateral	0.0 (0.0–0.0)	0.0 (0.0–0.0)	0.12

**Table 6:** Predictors of the volume of new ischemic lesions.

	Total Population			CAS			CEA		
	Beta	95% CI	*p*	Beta	95% CI	*p*	Beta	95% CI	*p*
Fazekas scale [12]	39.37	−24.47–103.20	0.22	60.34	−61.53–182.22	0.31	3.53	−35.90–42.96	0.85
Presence of chronic lesions	168.19	−74.18–410.55	0.17	319.82	−137.62–777.25	0.16	−39.55	−185.06–105.96	0.58
Volume of chronic lesions (mm^3^)	0.01	0.001–0.02	0.02	0.01	−0.002–0.02	0.12	−0.02	−0.16–0.11	0.71

## Data Availability

https://clinicaltrials.gov/ct2/show/NCT03840265 (accessed on 8 May 2023).
